# Outlining a novel psychometric model of mental flexibility and affect dynamics

**DOI:** 10.3389/fpsyg.2023.1183316

**Published:** 2023-12-14

**Authors:** Francesca Borghesi, Alice Chirico, Pietro Cipresso

**Affiliations:** ^1^Department of Psychology, University of Turin, Turin, Italy; ^2^Department of Psychology, Research Center in Communication Psychology, Universitá Cattolica del Sacro Cuore, Milan, Italy; ^3^Istituto Auxologico Italiano, IRCCS, Milano, Italy

**Keywords:** mental flexibility, affective states, core affects, psychometrics models, affect dynamics, Markov chain

## Abstract

Theoretically, affective states have always been conceived as complex phenomena enabling individuals to respond flexibly and dynamically to environmental demands. Methodologically, the novel field of Affect Dynamics has started to analyze affective states as inherently dynamic and interdependent phenomena by focusing on how and why they fluctuate over time. Fluctuations of affective states can also be conceived as a function of individuals’ ability to flexibly modulate their responses according to environmental demands. However, this ability has been sparsely investigated in different disciplines and domains, thus, engendering a plethora of terms and models. In this conceptual analysis, we first aimed to disentangle the puzzle of flexibility by outlining the distinctive cross-domain features of this concept, thus providing a novel comprehensive operationalization. We termed this novel unitary concept “mental flexibility,” the general ability to variably adapt to environmental demands. Then, we outlined the interplay between individuals’ mental flexibility and affect dynamics by proposing a novel psychometric model of affect dynamics, using Markovian chain.

## 1 Introduction

Affective states are inherently dynamic since they vary throughout the day, changing from moment to moment due to external events and individuals’ appraisals (Hamaker et al., 2015; Kuppens, 2015; Waugh and Kuppens, 2021; Schiller et al., 2022).

These emotional dynamics allow individuals to be more flexible, thus, adapting effectively to internal and external demands (Kuppens, 2015; Kishida and Sands, 2021; Ullah et al., 2021; Waugh and Kuppens, 2021). Hence, understanding the underlying mechanisms of affective dynamics is crucial for exploring novel trajectories for improving individuals’ well-being and health. In this conceptual analysis, we hold that one of the core mechanisms underlying affect dynamics is flexibility, as a cross-sectional property (i.e., affective, cognitive, physiological, behavioral) concerning the managing of affect over time effectively and quickly. First, we reviewed cross-domain evidence on flexibility as a property of different executive functions and processes. Second, we proposed a novel concept, mental flexibility, defined as individuals’ general ability to manage affective states at the cognitive, behavioral, physiological, and affective level (Mauri et al., 2010; Borghesi et al., 2023). Finally, we explained the interplay between flexibility and affective dynamics by introducing a novel psychometric model of affect dynamics.

### 1.1 Where is “flexibility”?

Try to imagine someone visualizing something extremely sad to keep from laughing in front of their hated boss. Now, consider when a person is immersed in a complex and confusing problem, which is suddenly resolved by shifting the focus from one aspect of the problem to another. Try to recall the last time you came up with a good novel idea very quickly. Perhaps you felt that your repertoire of thoughts expanded since you were able to include old ideas that were unexpectedly connected to the current topic. In an instant, a novel idea arose from the combination of distant ideas and the acquisition of a fresh perspective.

Throughout all these anecdotal instances, “flexibility,” as a general property of the human mind, was required and exhibited. Although all the above-mentioned examples can be related to scientific constructs of functions (e.g., emotion regulation, creative thinking, the shifting property of executive functions), a comprehensive scientific account of flexibility as a property of each function remains undeveloped. Several behaviors, for example, are considered flexible and operationalized as such (e.g., multitasking, novelty production, flexible problem solving), but no consensus has been reached on the scientific definition of an activity imbued with flexibility.

Scientifically speaking, flexibility remains an umbrella term or a property shared by some cognitive and emotional processes (executive function, emotion regulation, creativity, emotional intelligence) rather than a unitary concept. Therefore, the first research question we attempted to answer concerned the nature and structure of the construct of mental flexibility. Subsequently, the second applicative methodological question concerned the implementation of a psychometric model explaining and linking the construct of mental flexibility to affective states, their dynamism and temporal dimension.

## 2 From flexibility to *mental* flexibility: outlining a novel unitary concept

Generally, the term *flexibility* refers to several constructs with different operationalizations, depending on a specific domain of discipline (Cañas et al., 2006; Kashdan and Rottenberg, 2010; Kuppens, 2012; Barbey et al., 2013; Aldao et al., 2015; Dajani and Uddin, 2015; Goubet and Chrysikou, 2019; Uddin, 2021). Despite a wide array of approaches, it is possible to identify two distinctive and recurrent features associated with “flexibility” in different domains and disciplines: a *cognitive and psychological-affective component, defined as Cognitive Flexibility (CF) and Psychological Flexibility (PF)* (Southwick and Charney, 2012). Although cognitive and psychological flexibility emerged as two facets of the same “flexibility” concept, they have always been investigated separately.

Cognitive Flexibility (*CF*) falls under the broader category of executive functions (i.e., memory and attention) or processes necessary to control goal-directed behavior. In this domain, flexibility is the ability to adjust cognitive and behavioral strategies in response to changing contextual demands. For instance, a person who completes a task adapts to new and unanticipated environmental changes (Cañas et al., 2006; Elen et al., 2011; Kuppens, 2012; Dajani and Uddin, 2015; Uddin, 2021). Executive functions include three latent variables, described as a mental set shifting (‘shifting’), information updating and monitoring in working memory (‘updating’), and inhibition of prepotent responses (‘inhibition’), that moderately correlate with one another although they are separable (Miyake et al., 2000; Dajani and Uddin, 2015; Uddin, 2021).

Psychological Flexibility (PF) can be described as the ability to either alter one’s behavioral patterns; the ability or willingness to be in contact with our emotions, thoughts, or sensations (private events), both wanted and unwanted (Kashdan and Rottenberg, 2010; Cobos-Sánchez et al., 2020; Edwards and Lowe, 2021; Rizzo and Schwartz, 2021; Fang and Ding, 2022; Chong et al., 2023; Yang et al., 2023). It is defined as the ability to fully contact the present moment as a conscious human being, changing or persisting in one’s behavior while doing so serves valued goals (Kashdan and Rottenberg, 2010; Christodoulou et al., 2023). The Psychological Flexibility component includes a sub-process called Emotional Regulation Flexibility (ERF) (Aldao et al., 2015; Burton and Bonanno, 2016; Quattropani et al., 2022). Emotion Regulation Flexibility refers to the ability to implement and switch between emotion regulation strategies that are synchronized with contextual demands (Aldao et al., 2015; Goubet and Chrysikou, 2019; Cobos-Sánchez et al., 2020; English and Eldesouky, 2020; Gonzalez-Escamilla et al., 2022).

**Figure 1:**
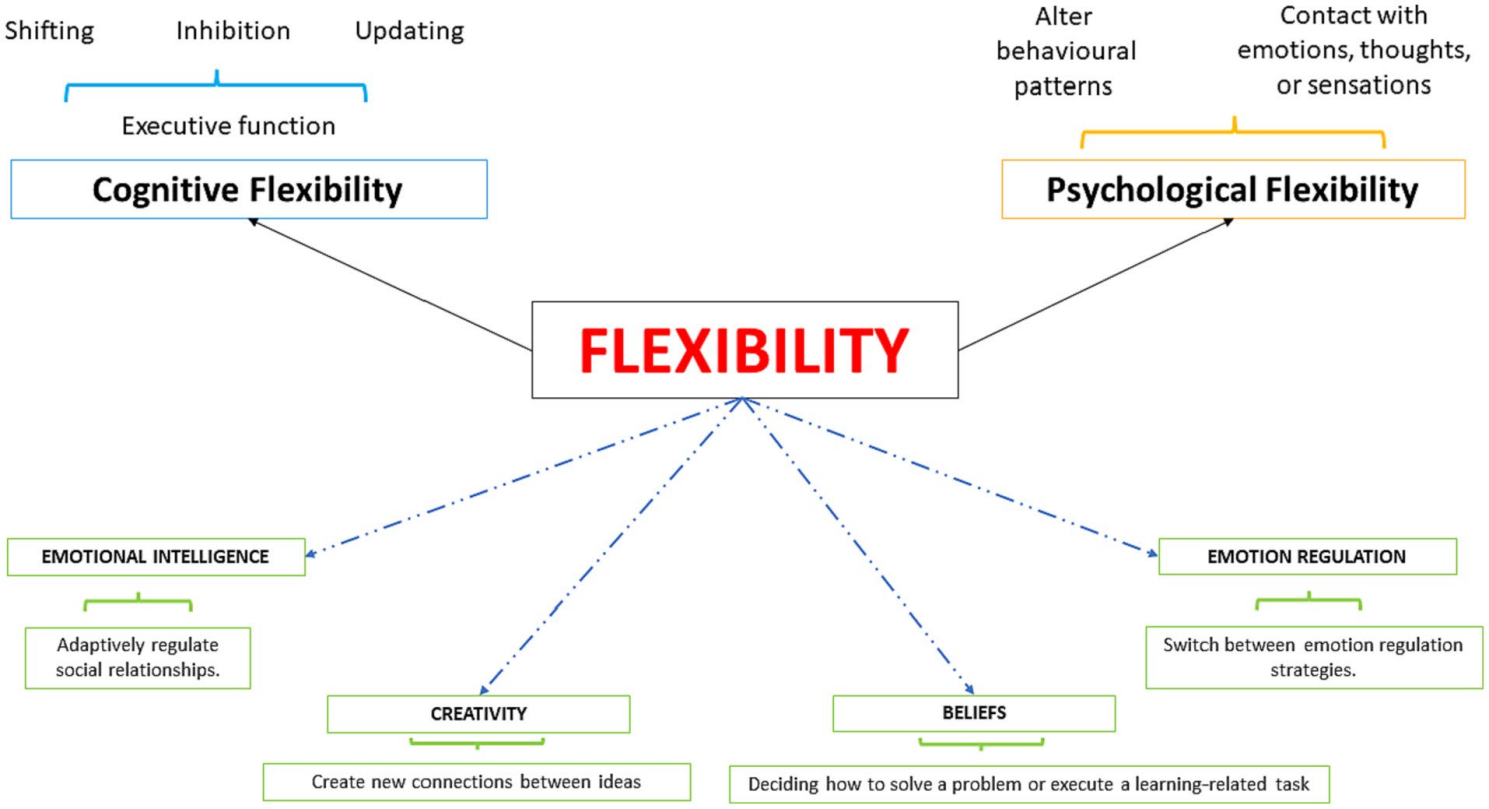
Components of flexibility.

Cognitive and Psychological flexibility have been examined in relation to other processes, e.g., Emotional Intelligence (EI), creativity, and beliefs (Nijstad et al., 2010; Elen et al., 2011; Arán Filippetti and Krumm, 2020; Cobos-Sánchez et al., 2020; Rizzo and Schwartz, 2021; Wu et al., 2021; Yang et al., 2023). Specifically, in these domains, flexibility emerged as the ability to adaptively regulate social relationships (Emotional Intelligence), create new connections between ideas (creativity), and influence beliefs. Beliefs are central to cognitive flexibility and relate to what individuals consider important, valid, and/or true (Elen et al., 2011). The relationship between epistemological beliefs – as a subset of general beliefs – and cognitive flexibility is of paramount interest: epistemological beliefs are a prerequisite for a cognitively flexible general attitude. Cognitive flexibility considers several pieces of information while deciding how to solve a problem or execute a learning-related task in various domains (Elen et al., 2011; Şahin et al., 2018). Emotional Intelligence (EI) is a type of social intelligence that involves the ability to monitor one’s own and other’s emotions, discriminate among them, and use the information to guide one’s thinking and actions (Mayer and Salovey, 1993; Mayer et al., 1999, 2004; Joseph and Newman, 2010; Fiori and Antonakis, 2011; Fossier, 2022; Kalyan et al., 2022). Here, flexibility refers to the ability to identify various emotions in different settings and adapt one’s emotional behavior to environmental demands. Creative thinking is operationalized in terms of flexibility since it is the ability to change strategy and to switch from one task to another but also the ability to abandon repetitive and usual patterns of thought to set out in new directions. Thinking flexibly is a valuable skill because it allows one to get out of a “thinking rut” to come up with a whole new idea (Nijstad et al., 2010; Benedek et al., 2014; Alhashim, 2020; Arán Filippetti and Krumm, 2020; Awe and Church, 2020; Figure 1).

All affective and cognitive dimensions of flexibility share the same feature, identifying as *adaptive* var*iability*. Var*iability* refers to the subject’s ability to feel, act, and understand things that happen to him or her from different points of view (Cheng et al., 2014; Hardy and Segerstrom, 2017). It can be described as a behavioral multidimensionality from which to observe and act. The ability to see a situation from different angles and act accordingly is often described as perspective-taking or perspective-shiftings (Lee et al., 2007). Similarly, *adaptivity* - the ability to adjust to different environments, situations, and challenges to reach a desired outcome – was often associated with resilience and resourcefulness (Froese, 2011; Macías-Escrivá et al., 2013). Some studies have also shown that *adaptivity* –the ability to respond adequately and quickly to environmental demands – positively correlated with achieving individuals’ goals over time (Wrosch et al., 2003; Aldao and Nolen-Hoeksema, 2012).

Specifically, at the behavioral level, *adaptivity* refers to the ability to *react* appropriately to environmental perturbations, whereas var*iability* refers to the ability to *choose* among the various views, emotions, and beliefs that a person holds (Paulhus and Martin, 1988; Macías-Escrivá et al., 2013; Cheng et al., 2014). Flexibility combines adaptive behavioral capacities with different strategies for dealing with variation. At the behavioral level, flexibility entails the ability to switch between different strategies in response to changing conditions and to modify behavior according to the situation. It involves adapting to different environments and situations, being open to new ideas and perspectives, and adjusting one’s behavior to different contexts. Flexibility connects the multidimensionality of affects and beliefs that characterize var*iability* with the adequacy and readiness that characterize *adaptive capacity*. Flexibility allows individuals responding to changing environmental conditions by adjusting their beliefs and behaviors quickly and effectively, by also safeguarding their core values and beliefs.

**Figure 2:**
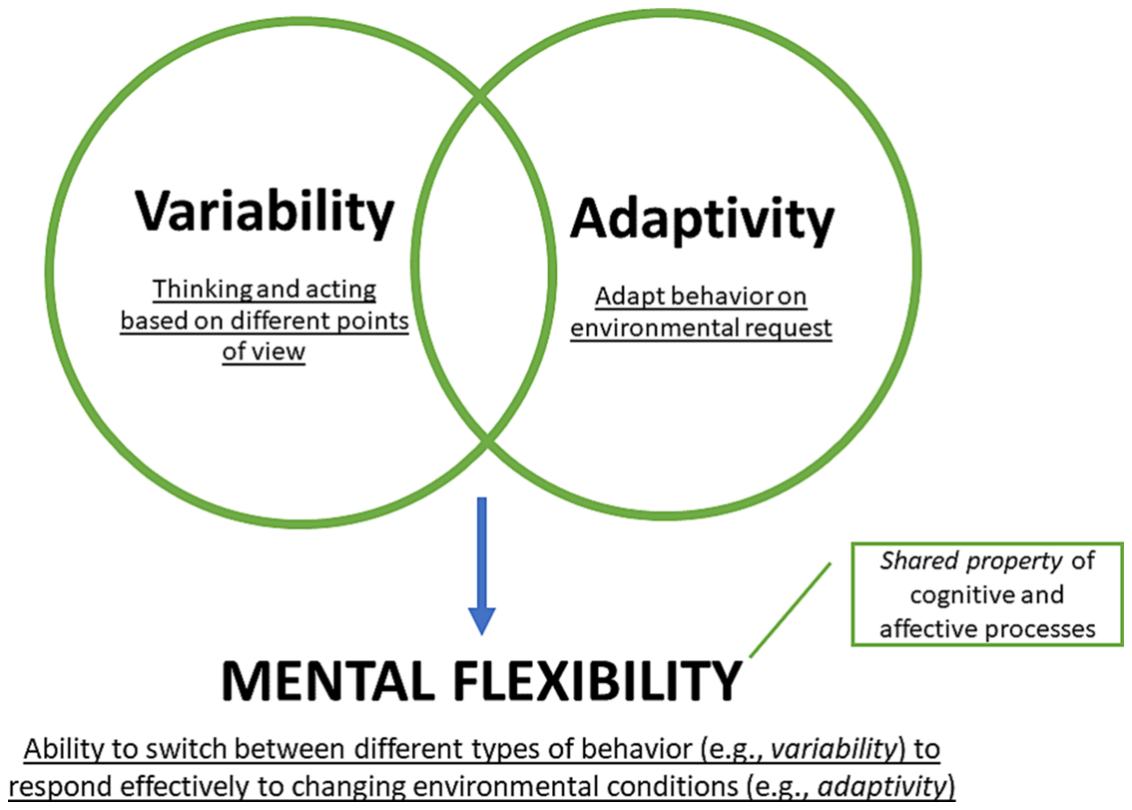
Flexibility as “adaptive variability” property.

Furthermore, it avoids *excessive variability*, leading to inconsistent or unpredictable behavior (e.g., indecision, inconstancy, impulsiveness). If an individual’s behavior varies widely in different situations, it may be difficult for others to know how to interact with this person or to predict how he or she will respond. This can lead to confusion or frustration in formal and informal relationships. Another potential dark side of variability is a lack of focus or direction. Individuals whose behavior constantly changes may struggle to identify and pursue specific goals or develop a clear sense of purpose. This can lead to a sense of aimlessness or lack of fulfillment (Lee et al., 2007; Müller et al., 2015). On the other hand, concerning the drawbacks of adaptability, flexibility can avoid the lack of *authenticity and accountability.* To adapt to different environmental and social demands, individuals may feel pressure to change their behaviors or values in ways that are not consistent with their true selves, so they are constantly adapting to new situations and shifting their behaviors or values without taking responsibility for their actions (Figure 2; O’Toole et al., 2020; Chen and Tang, 2022).

The flexible subject can adapt behavior effectively and quickly, acting according to environmental demands. In other words, flexibility emerges when cognitive and affective variability are in sync with changes in environmental demands. Thus, several cognitive and emotional processes exhibit flexibility in the form of its defining characteristic, “adaptive variability.” It could be interpreted as a shared property of various cognitive-affective processes, e.g., emotion regulation (Goubet and Chrysikou, 2019; Cobos-Sánchez et al., 2020; English and Eldesouky, 2020), emotional intelligence (Cobos-Sánchez et al., 2020; Rizzo and Schwartz, 2021; Wu et al., 2021), divergent thinking (Nijstad et al., 2010; Arán Filippetti and Krumm, 2020; Awe and Church, 2020), and beliefs (Cheng, 2001; Elen et al., 2011). This view would explain high correlations between cognitive flexibility and cognitive-affective processes. Moreover, Ionescu (2012) already hypothesized that cognitive flexibility, identified only as a neurological function related to executive functions, might be a shared feature of different processes.

Since flexibility is a property of different cognitive and emotional processes, we proposed adding the key adjective “mental” before it. This term allows us to position the definition and study flexibility unambiguously within the psychological domain without implying any connection with physics or materials chemistry. Henceforth, we would now refer to it as “mental flexibility” to describe a shared property characterized by complex affective-cognitive behavioral variability *(variability),* involving appropriate adaptation to environmental contingencies (adaptivity).

### 2.1 Measurements of mental flexibility

The lack of a unified conceptual definition of mental flexibility also has a methodological counterpart. Two main types of measurements can be used to assess flexibility: Direct Measurements (DM) and Indirect Measurements (IM), both referring to the ability to modify or shift between “cognitive sets” or strategies in response to changes in the environment (Bringmann et al., 2019). Measures called “direct” directly analyze the subject’s behavior in cognitive tasks, measuring attentional and memory processes (Cañas et al., 2006; Dajani and Uddin, 2015; Uddin, 2021), e.g., Wisconsin Card Sorting Test (Miles et al., 2021), Trail Making Test (Kortte et al., 2002), and Verbal and Semantic fluency (Kuppens, 2012). All of them are based on the assumption that transitioning from processing a series of letters to processing a sequence of numbers requires flexibility and inhibition, since switching the sequence of numbers implies blocking the sequence of letters. Indirect tests are based on individuals’ responses to the self-reported questionnaire, measuring the affective and psychological part of flexibility. The Cognitive flexibility scale (CFS) (Martin and Rubin, 1995), Cognitive Flexibility Inventory (CFI) (Dennis and Vander Wal, 2010), Cognitive Control and Flexibility in the context of Stress and Depressive Symptoms (CCFQ) (Gabrys et al., 2018), and Flexible Regulation of Emotional Expression (FREE) (Burton and Bonanno, 2016) are the most widely used scales. CFS measures communicative and emotional abilities, CFI measures interpersonal relationships, CCFQ assesses emotional coping based on stress and depressive symptoms, and FREE assesses flexible emotion regulation (see Table 1). Sample items include: “I approach the situation from multiple angles; I manage my thoughts or feelings by reframing the situation; I control my thoughts and feelings by putting the situation into context; I get rid of negative effects by changing the way I think about the situation.”

**Table 1:** Measurements of mental flexibility.

*Mental flexibility* measurements	
Direct measurements (DM) neuropsychological tests	Indirect measurements (IM) self-report questionnaires
*Trail-Making Test (TMT*): Executive function (attentional shifting and conflict monitoring) *Verbal fluency, phonemic*, *and semantic fluency* *Wisconsin Card Sorting test (WSCT):* Executive function (attentional shifting and conflict monitoring)	*Cognitive Flexibility Inventory* (CFI) (Dennis and Vander Wal, 2010): Flexibility in relationships *Cognitive Control and Flexibility Questionnaire* (CCFQ) (Gabrys et al., 2018): Flexibility in a stress situation *Cognitive Flexibility Scale* (CFS, Martin and Rubin, 1995): Flexibility in communication *Flexible Regulation of Emotional Expression Scale* (FREE) (Burton and Bonanno, 2016): Flexibility in emotion regulation

Recent meta-analysis articles showed that the two approaches (IM and DM) did not assess the same construct of flexibility, so self-report and neuropsychological tests of “cognitive flexibility” are not interchangeable evaluative tools (Fang and Ding, 2022; Howlett et al., 2022). However, the measurements share the multidimensional perspective: perceiving things from a different viewpoint, considering several points of view, identifying with multiple points of view, and acting in diverse ways with adaptive flexibility. However, no cognitive-affective questionnaire accounts for both components. Finally, central or peripheral neurophysiological investigation has also measured the affect and cognitive components of mental flexibility separately. Specifically, a previous study investigated central activations, characterized as executive functions, associated with cognitive flexibility (Badre and Wagner, 2006; Barbey et al., 2013; Dajani and Uddin, 2015; Uddin, 2021). A large body of literature on human functional neuroimaging studies using task-switching and set-shifting paradigms has emphasized the central role of the lateral frontoparietal network (L-FPN) and the mid cinguloinsular network (M-CIN) in supporting executive function and cognitive flexibility.

Furthermore, dynamic patterns and brain variability between specific networks have also been linked to cognitive flexible patterns. Dynamics between the default mode or medial frontoparietal network (M-FPN and the L-FPN) have been linked to cognitive flexibility (Douw et al., 2016). Brain variability increases during task performance compared with rest in younger and faster-performing adults, whereas older and slower-performing adults exhibit less differentiation in brain variability across experimental conditions (Garrett et al., 2013). Regarding Affective Flexibility, high Heart Rate Variability (HRV) is associated with higher emotional well-being (Kemp and Quintana, 2013; Beauchaine and Thayer, 2015), lower levels of worry and rumination (Ottaviani et al., 2016), lower anxiety, and better regulated emotional responding (Ochsner et al., 2009). Thus, individuals with higher HRV appear to better regulate their emotions (Appelhans and Luecken, 2006; Mather and Thayer, 2018).

Finally, even in measurements, mental flexibility dwells between *variability and adaptivity*: it emerges a complex pattern of behavioral variability, requesting emotional and cognitive adaptation to environmental demands.

## 3 Affect dynamics as a function of mental flexibility

Mental flexibility is a psychological construct referring to the individuals’ abilities to alter their states while dynamically adapting to different contexts and situations. Developing this skill is of the utmost importance when one must dynamically alter one’s state of mind in response to the obstacles of everyday life; such an adaptation can only be comprehended and investigated within the realm of emotions and feelings.

It is reasonable to suppose that mental flexibility and affective states are strongly related, and several studies have tried to explore this relationship (Aldao and Nolen-Hoeksema, 2012; Southwick and Charney, 2012; Gonzalez-Escamilla et al., 2022; Nahum et al., 2022; Borghesi et al., 2023). However, the dynamic nature of both processes complicates the analysis and requires systematization in the context of a psychometric model. Our study extended the current valence-arousal space to include flexibility, consistent with the related hypothesis, which proposed that it might be a determinant of affective dynamics more than any other psychological trait. Each affective state has a unique configuration of arousal-valence, but its expression and relationship with others may depend on mental flexibility levels.

### 3.1 Dynamic affective states

From a neuroscientific perspective, affects are multidimensional and polyhedric processes, entailing subjective, behavioral, vegetative, cognitive, and hormonal changes (Hamaker et al., 2015; Puccetti et al., 2021; Cipresso et al., 2023). Generally, there are three main theories, defined as Categorical, Dimensional, and Compositional (Schachter and Singer, 1962; Sloman et al., 2003). Categorical and Dimensional theories analyze each affective state as a unique expression of itself separately (Russell, 2003), while Compositional theories study affective states as continuous transitions from one to another.

Categorical theories consider each affect as an independent discrete entity and distinguish a small set of basic affects from a larger set of complex affects (Izard, 1984; Lazarus, 1991; Ekman, 1992). The basic affects are considered universal, innate, pancultural, evolutionarily ancient, shared with other species, and expressed by physiological and facial configurations with specific and distinct autonomic nervous system (ANS) patterns (Ekman, 1992). On the contrary, complex affects are learned and shaped by evolutionarily new social and cultural factors. They are more evident in humans and normally expressed through combinations of response patterns that characterize basic affects. Complex affects, influenced by the language, emerge relatively late in development. However, human beings usually do not experience affects as specific and discrete entities but as ambiguous, blurred, and often overlapping experiences (Ekman, 1992).

**Figure 3:**
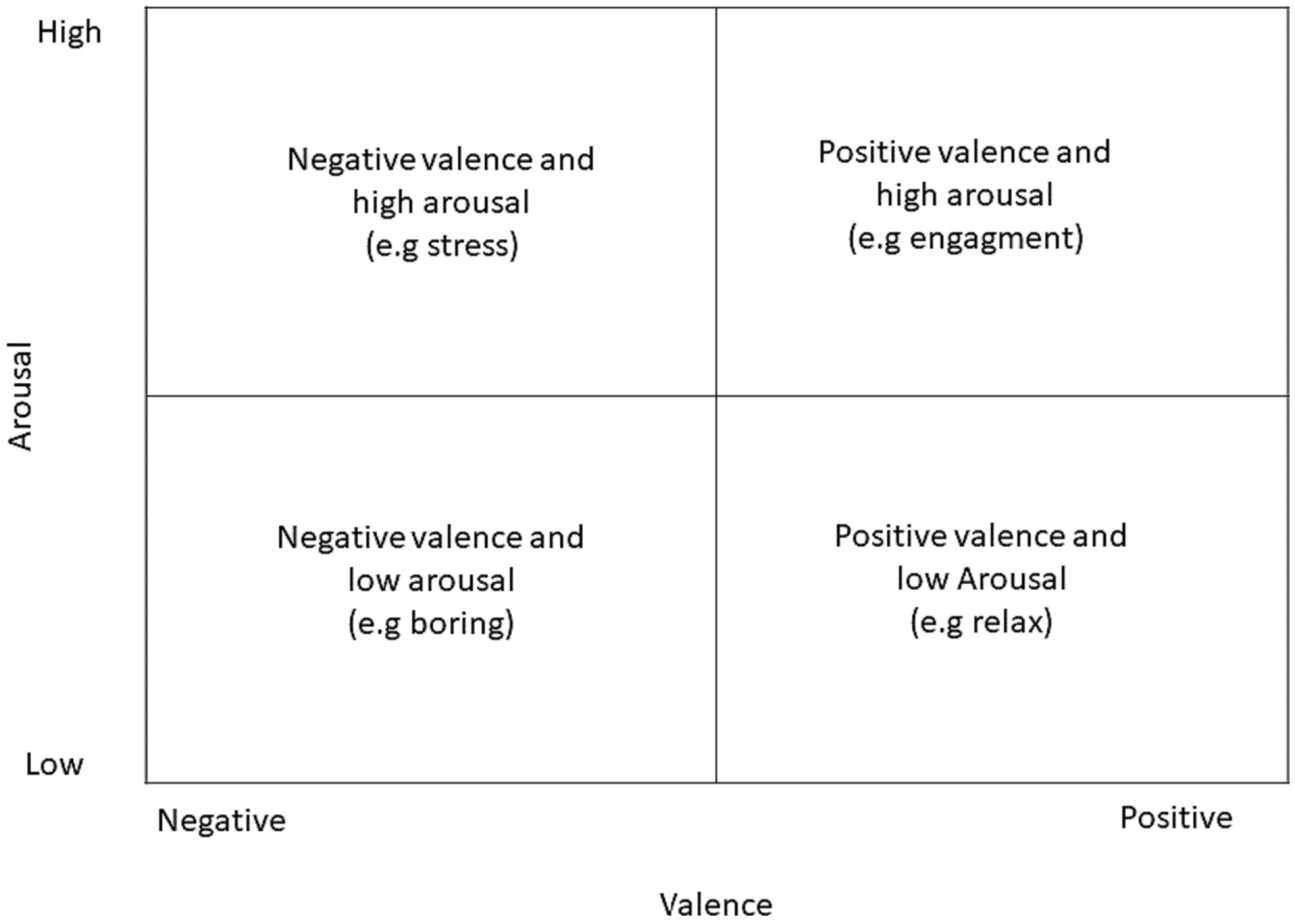
Dimensional model of affect.

In this regard, dimensional theories consider each affect as a point along a continuum between two fundamental axes: arousal and valence dimension (Figure 3). The arousal dimension is considered the neurophysiological activation and valence as its relative, subjective intensity and pleasantness of affects or feelings (Russell and Barrett, 1999; Russell, 2003, 2017).

**Figure 4:**
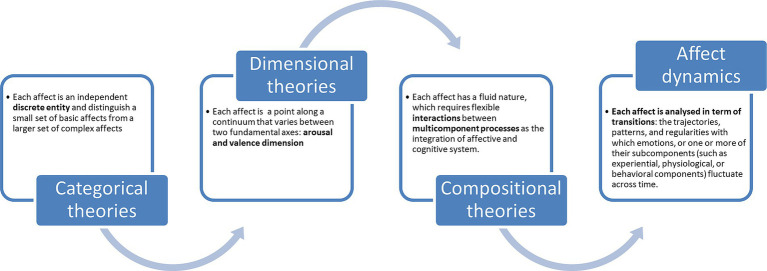
Summary of affective states models.

The subject can identify and differentiate between the strength of activation and the pleasantness of affects; the subjective experience of affect is only defined as an emotional sensation. Affects are thought to be static and distinct from one another while being complex dimensional states (Posner et al., 2005; Russell, 2017). His fluid character, necessitating the flexible transition between several emotional states, is rarely examined even though affect is seen as stable. For this reason, a novel approach to affect classification has been introduced, which relies on the theories of compositional processes (Scherer, 2009). These theories offer a third innovative and interesting way of classifying those accounts for a multidimensional vision of affect classification. These theories aim to capture affect’s fluid character rather than seeing them as static states. To do this, they propose adaptable interactions among the multicomponent processes that underlie the integrated emotional and cognitive system. Compositional theories highlight the cognitive processes involved in assessing the affective significance of events and the relationship between the assessment’s outcome and behavioral and physiological responses. Scherer (2009), one of the most important authors, argued that dynamic affective processes are based on an individual’s subjective appraisal of significant events. For the first time, compositional theories focused on the dynamics of affective states linked to the influence of higher cognitive processes. Fluid transitions result from rewriting and re-evaluating the environment where one feels the affect (Figure 4).

*Affect dynamics*, representing the final frontier of affective states, encompasses and outperforms compositional theories (Waugh and Kuppens, 2021). It considers affective state transitions in terms of their trajectories, patterns, and regularities with which emotions or one or more of their subcomponents (such as experiential, physiological, or behavioral components) fluctuate across time, their underlying processes and downstream consequences (Hamaker et al., 2015; Waugh and Kuppens, 2021). Each affective state has a unique configuration of arousal-valence, but its temporality characterizes its expression and relationship with others. The affective states are defined as situational and variable states able to change flexibly and adapt to situational triggers or as strict and rigid states in which no change is possible. Temporal alterations in processes that underlie emotional response determine emotion dynamics.

### 3.2 Being flexibly dynamic: a novel psychometric model of affect dynamics

Flexibility, or adaptive variability, may explain affective states’ transitive dynamism. Our **hypothesis** draws from compositional theories (Scherer, 2009), trying to give a more precise methodological and definitional classification of affective-cognitive processes. The goal is to create a multidimensional affective dynamic model, in which the dimensional arousal-valence model is integrated with a third cognitive-affective dimension that forms the complexity and the temporality of affective states.

Here is an anecdotical example from everyday life. Mary has a high level of mental flexibility, as evidenced by her ability to regulate her emotions, relationships, and communication and closely monitor and control daily life events; however, she was reprimanded for poor work performance today. This has undoubtedly shaken her emotional state, moving her from relaxation to stress. Unlike people with limited mental flexibility, she could experience both affective states, alternating her mood according to circumstances. Less flexible people, on the other hand, suppress one of the two affective states, being conditioned only by one. Additionally, Mary saw the stressful situation as an opportunity to learn. She did not consider her failure as a personal or total failure. Lastly, Mary could look for new and creative solutions. She has an affective dynamism that allows her to switch among different affective states adaptively taking advantages also from negative situations.

**Figure 5:**
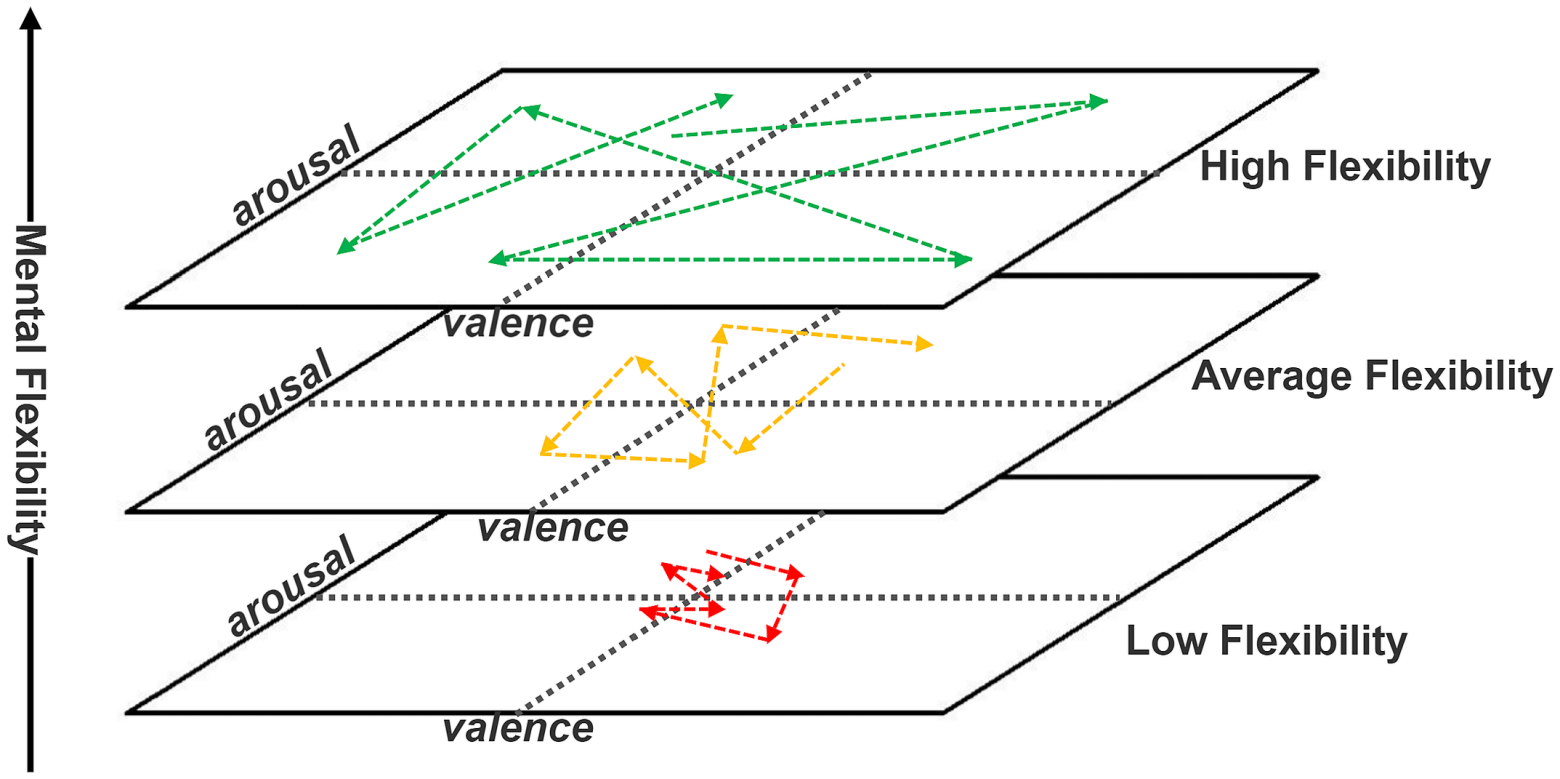
Model of affect dynamics, influenced by mental flexibility.

According to our theory, affective-cognitive properties of mental flexibility support and go beyond the traditional dimensional description of affect. This article proposed a new psychometric three-dimensional modeling of affect measurement or measuring behavioral, subjective, and neuropsychophysiological changes associated with an affective episode. This represents an intersection between the consolidated two-dimensional arousal-valence model and a third component defined as mental flexibility, including high-level cognitive processes associated with the cognitive-affective sphere, such as affect regulation, divergent thinking, and executive function (Bonanno et al., 2004; Kashdan and Rottenberg, 2010; Nijstad et al., 2010; Arán Filippetti and Krumm, 2020; Cobos-Sánchez et al., 2020; Edwards and Lowe, 2021; Rizzo and Schwartz, 2021; Wu et al., 2021). Mental flexibility could have a possible top-down influence allowing flexible interaction and transition between the various affective states. Each affective state has a unique configuration of arousal valence, but its expression and relationship with others may depend on mental flexibility levels. We expected to find a statistically significant difference in transition movements between affective states based on the level of mental flexibility: those with high mental flexibility would be expected to move more flexibly and variably between the various affective states (as exemplified by the green and yellow arrows), whereas those with low mental flexibility would be expected to move between the various affective states invariably, with no significant differences (red arrows) (Figure 5).

Flexible and fluid emotional transitions were used as synonyms for *thoughtful smoothness*. The emotive experience is a complicated activation pattern. Its subjective and physiological activity (valence and arousal) may be regarded as a component of a higher cognitive system that continually evaluates settings and external and internal inputs and adapts and changes affective behavior. Different emotional stimuli continually excite a subject in a real or experimental situation, generating multiple affects: after prioritizing affective activations, the person can move freely on the generated affective states. As a result, the subject’s movement flexibility, enabled by high-level flexible processes, allows him to adaptively change his emotional responses to affective inputs without being trapped in dysfunctional affective states. As a result, adding mental flexibility enables us to categorize emotional states: flexible alterations enable us to comprehend the changing appraisal of affect.

Those individuals with limited mental flexibility are anticipated to exhibit less adaptive flexibility, resulting in a persistent activation of the same affective state, as mental flexibility **is anchored** and influenced by the **previous activation** when the starting point is an affective state with a positive or negative valence or high and low arousal (Figure 5).

People with low mental flexibility often struggle to transition from one emotional stimulus to another because they tend to get “stuck” in a particular emotional state. They are more inclined to fixate on a specific emotion or experience, finding it difficult to open up to new feelings. This lack of flexibility hinders their ability to explore a wider range of emotions and limits their capacity to adapt to different situations.

On the other hand, highly flexible individuals can move more easily from one emotional state to another. They are open to emotional exploration and can regulate their emotions more fluidly. Their minds can adapt quickly to different circumstances, enabling them to experience greater emotional variability. This mental flexibility allows them to effectively navigate emotional challenges and benefit from a broader range of affective experiences.

The proposed model recognizes that mental flexibility plays a crucial role in shaping an individual’s ability to adapt cognitive processes in response to changing emotional stimuli. It also acknowledges that affect dynamics, which refer to the temporal fluctuations in emotional states, can be influenced by own’s cognitive flexibility (Borghesi et al., 2023). By combining these measures, we can explore how changes in cognitive flexibility may impact affective experiences and how variations in affect dynamics may influence cognitive processes. For affect dynamics, two types of statistical indices are prominent: those based on variability (e.g., Standard Deviation, Root Mean Square of Successive Differences, i.e., RMSSD, Teager-Kaiser Energy Operator, i.e., TKEO) or those based on inertia (e.g., Mean as a trait characteristic, Inertia indices, and Autocorrelation). These indices are commonly used in multivariate models, such as panel data in time series analysis or dynamic multilevel modeling. On the other hand, flexibility does not have specific indices as it depends on self-report assessments or neuropsychological tests. To our best knowledge, currently, there is no valid test that captures both the cognitive dimension of executive functions (cognitive flexibility) and the affective dimension (affective flexibility) simultaneously.

### 3.3 Model definition and initial calibration

Here we propose a psychometric model that integrates measures of flexibility with those of affect dynamics. This model aims to capture the dynamic relationship between an individual’s cognitive flexibility and their emotional experiences. By incorporating both constructs into a unified framework, we sought to gain a more comprehensive understanding of how they interact and influence each other.

We present a calibration conducted as part of an ongoing experimental investigation. The study proposed involved an exploratory modelling of the relationship between mental flexibility and affective dynamics, through Markovian Chain. The design experiment consisted of two parts: a testing phase to assess levels of mental flexibility and a behavioural-physiological phase involving the administration of emotion-inducing stimuli to elicit and measure affect dynamics. The self-report measures included the *Cognitive Flexibility Inventory* (CFI) (Dennis and Vander Wal, 2010; Portoghese et al., 2020) and the *Cognitive Flexibility Scale* (CFS) (Martin and Rubin, 1995).

**Figure 6:**
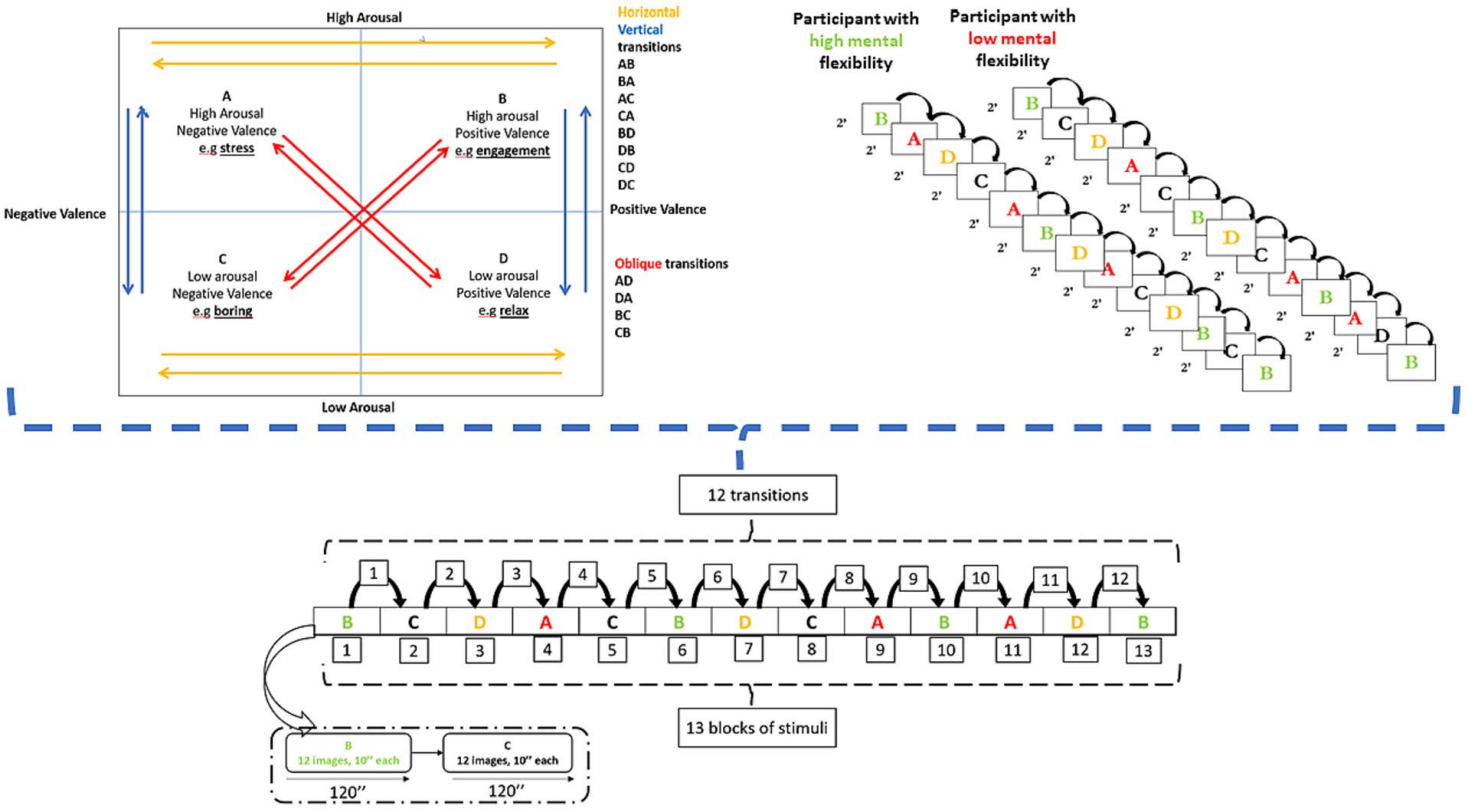
Experimental design (left side) and two examples of block sequences: High flex vs. Low Flex (right side).

The selected emotion-inducing stimuli were obtained from the *International Affective Picture System* (Lang et al., 1997) and were organized into 13 blocks of different valence and arousal levels, each lasting 2 min (with 10 s per image), resulting in a total of 12 transitions, as illustrated in Figure 6. Each participant had a randomized sequence of 13 blocks: here, we presented the sequence of participants with high scores of mental flexibility and low flexibility (Figure 6).

During the stimulus presentation, we collected several physiological signals, including zygomatic major and corrugator supercilii facial electromyography (f-EMG), Skin Conductance (SC), Respiration (RESP), and Blood Volume Pulse (BVP) form with it is possible to extract several indexes of Heart Rate Variability (HRV). For the initial calibration, we considered only the corrugator supercilii f-EMG as expressions of emotional valence, as indicated in the literature (Mauri et al., 2010; Cipresso and Immekus, 2017; Cipresso et al., 2019; Kehri et al., 2019; Mancuso et al., 2022).

We considered, in an exploratory and illustrative manner, two “extreme” participants: one with very high levels of mental flexibility (scores of CFI, CFS >6) and one with very low levels of mental flexibility (scores of CFI, CFS < 3) (Figure 6).

The methodological aim was to demonstrate how different levels of flexibility corresponded to different variability in affective transitions between blocks representing all the 12 transitions possible. Our aim was to employ a new psychometric approach based on Markov chains to analyse the variability of affective transitions, as reported by Cipresso et al. (2023). Markov chains provide a mathematical framework to model and analyse dynamic processes among states, where a future state depends solely on the present state. This property is particularly relevant for studying affect dynamics, as it allows us to capture the temporal dependencies and transitions between affective states. Our experimental design enables us to calculate and assess the variability across different affective transitions, which can be normalized and incorporated into the Markov chains. By doing so, we effectively capture the probability of transitioning between blocks while accounting for the total variability.

This approach aligns well with the core characteristic of Markov chains, where future states are influenced only by the current state, enabling us to model and understand the affective dynamics within our experimental paradigm. Hence, the initial challenge was to determine how variability could be calculated and develop a corresponding relative index that aligns with the characteristics of Markovian chains. Indeed, in Markov chains, it is a requirement that the rows of the transition matrix sum to 1, because the transition probabilities in each row represent the probabilities of transitioning from one state to all possible states in the system.

Firstly, we calculated standardized variability measures, based on the inverse of Noise to Signal of the corrugator supercilia f-EMG quantifying the ratio between the unwanted noise or interference in a signal and the desired signal itself. In this case, it would correspond to the reciprocal of the ratio between the absolute mean of the signal and the variability of the signal itself. In summary, the reciprocal of Noise to Signal, as an index of standardized variability, is useful to describe physiological signals, particularly in the field of EMG, enabling comparisons and interpretations that are independent of specific measurement scales or units (Reaz et al., 2006). It consisted of ratio of standard deviation of transition and his absolute mean. If the coefficient is close to zero, it indicates that the standard deviation is relatively small compared to the mean, implying minimal relative variability among the data. Conversely, as the coefficient increases, it exemplifies greater variability in the data.

**Figure 7:**
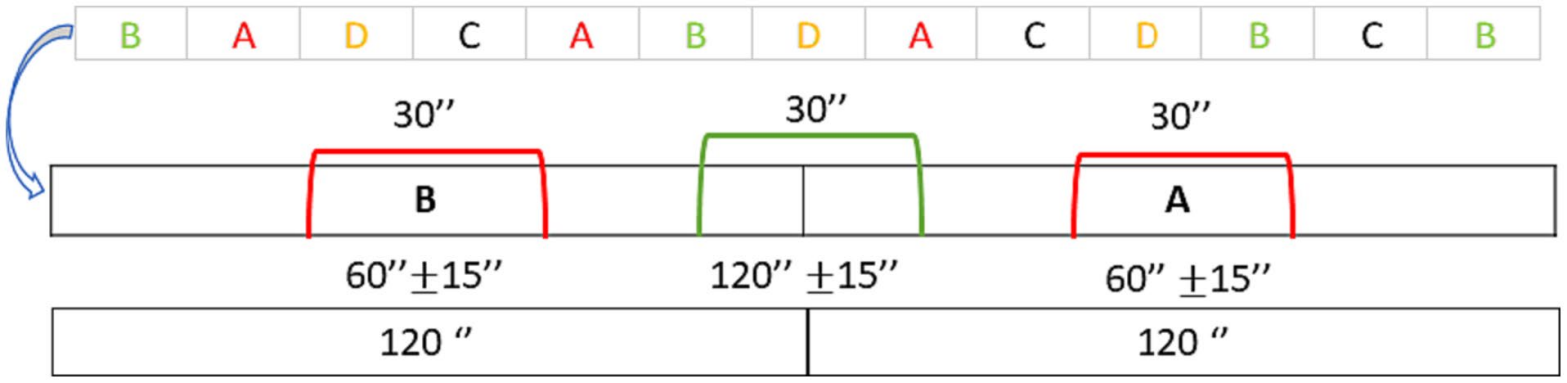
Timing of blocks: timing of transition between states (green curly bracket) and timing of self-state transition (red curly bracket).

For each transition, the standardized variability index was calculated, considering the 30-s interval spanning across the blocks (120″ ± 15″) (Figure 7). However, in the Markov matrix, it was also necessary to consider state indices for each block, specifically the transition of state A with itself. State transitions (the way in which you remain in the same state in which you are) were measured as the middle 30″ of each block (45″-75″), representing the most descriptive and informative portion of the elicited affective state, preceding the transition to the next block and following 60″ after the previous one (Figure 7).

This aligns well with Markov chains, where each future state depends solely on the present state.

**Figure 8:**
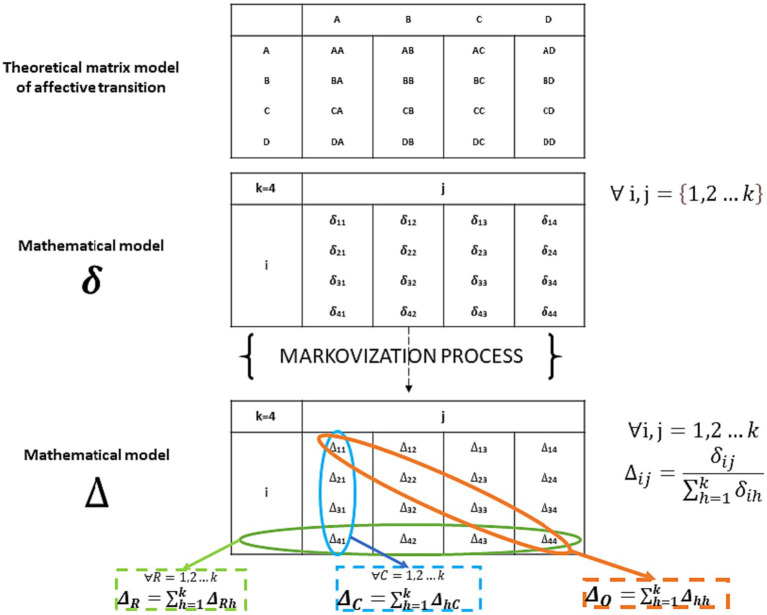
Structure of transition matrix and delta index.

Once the 16 variability indices ( δ ) were calculated for each subject (12 for transitions between blocks +4 for state transitions), they were normalized within the Markov matrices as expressions of transition variability probabilities from one block to another, relativizing them to the total variability of transitions, as exemplified in Figure 8 ( Δ ). The normalization of transition indices is necessary because in Markov matrices, the sum of transitions is equal to 1. Therefore, we considered and validated a new index that expresses the relative magnitude of a transition’s variability compared to the total row variability of all transitions. This index allows us to assess how much a specific transition contributes to the overall variability in the transition matrix (Figure 8). We called it Δ index.

Indeed, δ index expresses the transitions between states in probabilistic terms, linking them to standardized variability ( Δ ). As a result, the main diagonal of the matrix (lowest part of Figure 8) represents the probabilities of staying in the same state, indicating stability. On the other hand, the off-diagonal elements represent the probabilities of transitioning between different states, indicating changes or variability between states. This probabilistic representation provides valuable insights into the stability and variability of state transitions within the system being studied. As an example, among the off-diagonal indices we can calculate the sums per column and per row, which give us the information of the % of being source (row) of a state and receiver (column). For example, it might be interesting to investigate whether a subject has an easier time making transitions if they start from an affective state (e.g., relaxation/joy), or which state the subject is most likely to end up in (lowest part of Figure 8).

For Markov chain calculation we used Matlab R2023a. For the initial states (S0), equal probabilities of transition were assumed for both subjects [p(X_A-B-C-D_ = 0.25)], however a different distribution of initial states does not affect the steady states or the transition matrix.

**Figure 9:**
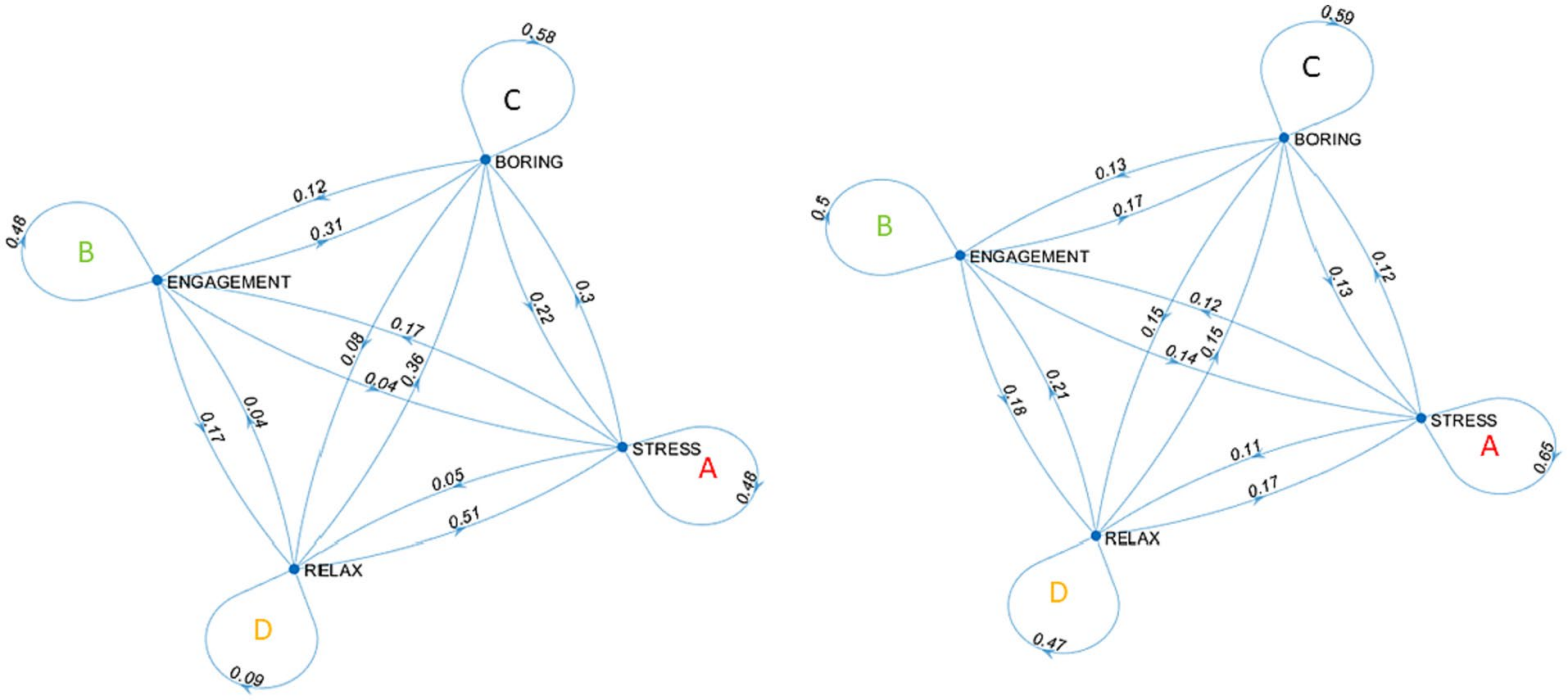
Graph of the transition matrixes: subject with High Flexibility vs. subject with Low Flexibility.

In the Markov process, the input structure consists of the transition matrix, and Figure 9 represents it in the form of the mathematical structure known as a graph (in particular, a weighted directed graph with transition probabilities as weights).

Instead, the output structure is the *steady state vector*, referring to the long-term equilibrium distribution of probabilities for each affective state, denoted by A, B, C, and D blocks in this case. For this example, we chose not to present the other possible indices (i.e., sum of row, column and diagonal) but to refer only to the steady state as the primary output of the Markov matrix. It represents the probabilities of being in each state over an extended period, irrespective of the initial state:


$$\pi =\left[\pi_{1},\pi_{2},\ldots ,\pi_{i},\ldots ,\pi_{N}\right]\text{,}$$

where πᵢ represents the stationary probability associated with state *Sᵢ*.

The steady state is influenced by the transition probabilities between states and reflects the relative stability or dominance of variability of each affective state in the system. It provides valuable insights into the prevailing variability of affective patterns and their probabilities in the long run. It is calculated after five steps. Here an illustrative example to calculate steady state, after five steps for the two participants (Table 2).

**Table 2:** Steady state calculation of high flexibility (up) vs. low flexibility (down) participant.

Step	A (Stress)	B (Engagement)	C (Boring)	D (Relax)	Formula
S0	0.25	0.25	0.25	0.25	Initial State
S1	0.31	0.2	0.39	0.1	S0 × P = S0 × P1
S2	0.29	0.2	0.42	0.09	S1 × P = S0 × P2
S3	0.29	0.2	0.42	0.09	S2 × P = S0 × P3
S4	0.28	0.2	0.43	0.09	S3 × P = S0 × P4
S5	0.28	0.2	0.43	0.09	S4 × P = S0 × P5

Step	A (Stress)	B (Engagement)	C (Boring)	D (Relax)	Formula
S0	0.25	0.25	0.25	0.25	Initial State
S1	0.27	0.24	0.26	0.23	S0 × P = S0 × P1
S2	0.28	0.24	0.26	0.22	S1 × P = S0 × P2
S3	0.29	0.23	0.26	0.22	S2 × P = S0 × P3
S4	0.29	0.23	0.26	0.21	S3 × P = S0 × P4
S5	0.29	0.23	0.26	0.21	S4 × P = S0 × P5

To accurately interpret the steady state vector, it is essential to commence with a visual inspection of the transition matrix. The diagonal element in the transition matrix represents the probabilities of variability of staying within the same affective state. For the flexible subject, we observe the following diagonal probabilities: 0.48, 0.48, 0.58, and 0.09. These values indicate that the flexible subject has relatively lower probabilities of transitioning to the same affective state, suggesting a higher degree of extra-state variability and flexibility. Lower probabilities of variability are observed in remaining in the state of relax (0.09), indicating that once in the relax states flexible subject has no reason for changing since a positive situation, and this is part of its own mental flexibility. On the other hand, the low flexibility subject exhibits the following diagonal probabilities: 0.65, 0.50, 0.59, and 0.47. These values imply higher probabilities of remaining within the same affective state for this subject, indicating a more limited range of affective transitions and reduced extra-state variability. The subject with low flexibility appears to be more variable in remaining within a state and less variable in transitioning between other states. One hypothesis could be that low levels of flexibility restrict the ability to move freely between states, leading to a tendency to remain in the same state. This contrasts with high levels of flexibility, where transitions between different affective states are more variable, while transitions within the same state are less variable (Table 3).

**Table 3:** Transition matrix and steady state of participant of high flexibility and low flexibility.

High flexibility					Low flexibility				
	Stress	Engagement	Boring	Relax		Stress	Engagement	Boring	Relax
Stress	0.48	0.17	0.30	0.05	Stress	0.65	0.12	0.12	0.11
Engagement	0.04	0.48	0.31	0.17	Engagement	0.14	0.50	0.17	0.18
Boring	0.22	0.12	0.58	0.08	Boring	0.13	0.13	0.59	0.15
Relax	0.51	0.04	0.36	0.09	Relax	0.17	0.21	0.15	0.47
									
Steady state	0.28	0.20	0.43	0.09	Steady state	0.26	0.29	0.22	0.22

These results are confirmed by the final steady-state vector, considering 5 iterative steps. The steady state refers to the stable or equilibrium states within the Markov chain, where the probabilities of transitioning between states remain constant over time (Table 3). Those with higher levels of flexibility appear to have a greater likelihood of demonstrating variability in negative affective states (0.29 for the boring block and 0.43 for the stress block) in the long term. Interestingly, Engagement that had manifested a higher trait state appearing to be rigid on that state (0.48), changed a lot in the steady state (to 0.20), highlighting how flexible subject in the long run adapts the positive emotions lowering the variability of this positive state, thanks to the mental flexibility. Conversely, the steady states of individuals with lower mental flexibility appear to be substantially equiprobable, with the highest peak observed for the stress block (0.29). The equiprobability among the variabilities of affective states may be linked to the high scores on the diagonal of the transition matrices: low levels of flexibility may imply higher levels of variability in state-to-state transitions rather than transitions to other states, thereby reducing the probabilities of variability associated with steady states. Conversely, in a subject with high mental flexibility, the steady states are significantly different from each other, indicating how the probabilities of change vary across different affective states. It is important to note that the considerations made so far only pertain to variability related to corrugator supercilii facial electromyography (f-EMG), and more comprehensive studies will consider other physiological responses such as comparison with zygomatic major f-EMG, respiration, and heart rate variability indices, while also considering a consistent sample size.

## 4 Conclusion

Affective states have been often studied statically and bi-dimensionally as if they were independent of one another. Adding a third dimension of mental flexibility to Russell’s bi-dimensional arousal-valence model gives them dynamism and adaptability. In this context, affect states are seen as interconnected, complicated occurrences, and mental flexibility explains their intersection and transition. Those with high mental flexibility go from one emotional state to another in a variable manner. They can experience various affective states and may contextualize their experiences based on environmental demands. Consequently, mental flexibility, or my capacity to adjust to variation, influences my affective states. This concept has theoretical, methodological, behavioral, and neurophysiological applications.

Inserting mental flexibility in affect dynamics is a complex psychometric problem. The effect dynamic is exemplified by variability and change, best shown through mental flexibility. At the computational level, adapting a static model, such as arousal-valence, into a dynamic model is not straightforward. We usually analyze affective dynamics by looking at emotional/affective state changes over time; thus, it’s natural to conceive of time as the cause. However, time does not cause anything by itself. Rather, other causal agents of affective dynamics occur over time, and it is only our perception that time is the cause of these changes. Hence, mental flexibility could be the focus of the current affect dynamics research utilizing longitudinal and dynamic models (Baumann and Kuhl, 2005; Audet and Lefebvre, 2017; Kishida and Sands, 2021; Lazarus et al., 2021; Vanhasbroeck et al., 2021; Cipresso et al., 2023). Hamaker et al. (2015) presented an up-to-date summary of statistical and mathematical modeling tools that have or are being developed to evaluate intensive longitudinal data relevant to affective sciences concerns. In general, the most used methodologies are time series analysis and multivariate analysis, incorporating cross-lagged and panel data (Kuppens, 2015; Kishida and Sands, 2021; Vanhasbroeck et al., 2021; Waugh and Kuppens, 2021). In our conceptual design, we included a proposal for psychometric modeling using Markov chains. This type of analysis falls under *state transition models*, considering the probabilities or patterns of transitioning between specific affective states, providing insights into the temporal dynamics and underlying processes of affective fluctuations. Markov chains allow for the modeling of sequential dependencies and transitions between different affective states. This is crucial in capturing the temporal dynamics of affect, as it acknowledges that the current affective state is influenced by the previous state. Secondly, Markov chains provide a probabilistic framework to estimate the likelihood of transitioning between different affective states. This enables the quantification of transition probabilities, which can reveal patterns and tendencies in affective dynamics over time. Moreover, Markov chains offer a flexible and interpretable approach to analyzing affect dynamics. By representing affective states as discrete states in the chain, it becomes easier to interpret and compare different affective patterns and transitions. As an example, we considered two subjects, one with high flexibility and the other with low flexibility levels and exposed them to emotional images divided into 13 blocks of arousal and valence. We calculated a standardized variability index ( δ ) of corrugator supercilia f-EMG on the transitions and incorporated it into the Markov chain ( Δ ). The two transition matrices reveal that higher levels of flexibility are associated with a higher probability of physiological variability in transitions between blocks. Conversely, individuals with lower flexibility levels seem to have a lower likelihood of variability in transitions with other affective states, remaining fixed in a particular affective state. This finding supports the hypothesis that higher levels of flexibility correspond to a greater ability to vary and move between different affective states, while lower levels correspond to affective stability. Future studies should consider different physiological measures (e.g., respiration, heart rate variability), various indices (e.g., inertia index, entropy index), and consistent sample sizes when investigating affect dynamics.

This psychometric model can be adapted behaviorally and neurophysiologically to healthy subjects and patients. In particular, therapies such as Acceptance and Commitment Therapy (ACT) and Cognitive and Behavioural Therapy (CBT) seem to be connected to and influence the concept of flexibility (Audet and Lefebvre, 2017; Hardy and Segerstrom, 2017). Flexibility in the ACT model refers to being aware of thoughts and emotions in the current time without defense and continuing or altering actions to achieve key interests and objectives (Kashdan and Rottenberg, 2010; Pennato et al., 2013; Soares et al., 2023; Tan et al., 2023). Although CBT does not explicitly discuss flexibility as an aim of treatment, flexibility is such an integral part of psychological functioning that it is almost inevitable that it will, in some way, be affected. CBT helps patients attain valued wishes and form happy attitudes by strengthening positive motivation and interpersonal communication via therapist-patient interaction, leading to immediate behavioral objectives. Wang et al. (2023) showed that CBT could change psychological problems by altering patients’ views and attitudes towards themselves or things, which also applies to the elderly with cognitive decline. Furthermore, in this context, both the DSM-5-TR and the U.S. National Institute of Mental Health advocate for psychiatrists to assess flexibility in a dimensional manner rather than relying on categorical evaluations. The Research Domain Criteria (RDoC) approach emphasizes that flexibility dimensions can extend beyond traditional diagnostic categories, urging the integration of diverse data levels, spanning genomics, neural circuits, and behavior, including self-report measures such as participant-filled questionnaires. Cognitive and emotional rigidity or inflexibility represents commonly observed characteristics across various mental illnesses, particularly in clinical conditions manifesting early in life, such as Parkinson’s Disease (PD) and Anorexia Nervosa (AN), depression and bipolarism (Pantelis and Andrewes, 1995; Cools et al., 2001; Díaz-Santos et al., 2015; Pietschnig et al., 2016; Alzaid et al., 2020). Recent research has identified a correlation between motor stiffness in PD and executive processes (attention shifting and conflict monitoring), processes known to be influenced by cognitive procedural rigidity (Cools et al., 2001; Díaz-Santos et al., 2015; Alzaid et al., 2020). Conversely, AN is marked by emotional rigidity and challenges in transitioning between cognitive or affective states (Friederich and Herzog, 2011; Abbate-Daga et al., 2014; Halls et al., 2023). Mood disorders such as anxiety, depressive, or bipolar disorders are characterized by unique behavioral, cognitive, and affective rigidity (Audet and Lefebvre, 2017; Gabrys et al., 2018; Bosley et al., 2019; Edwards and Lowe, 2021). Subjects appear anchored in their affective states, with low variability either within or toward other states. Even should they succeed in affect passage, the transition turns out to be abrupt and uncontrolled. Investigating mental flexibility in terms of assessment and rehabilitation, may be of help in identifying the most impaired cognitive (set-shifting) affective (dynamic and transitions) areas.

Finally, future studies must consider dynamic stimuli to study affect dynamics. This kind of affect induction is quite difficult because it requires a dynamic change from one stimulus to another along a continuum that is difficult to elicit with videos and impossible to elicit with photos. In this sense, using classic stimuli in the valence-arousal model (e.g., International Affective Picture System [IAPS]) would be ineffective (Lang et al., 1997). Virtual Reality (VR) could be an interesting future step to examine the physiology and behavior associated with the transition between distinct emotional states (Cipresso et al., 2019). Virtual environments can be modifiable and continuous in eliciting emotions, where the same environment can change and elicit different affective states. Furthermore, VR allows for the development of interactive environments where individuals can engage, act, and perform actions. This enables the creation of situations that require individuals to demonstrate their flexibility. Utilizing immersive tools such as VR or 360-degree videos facilitates the creation of behavioral tests on mental flexibility, augmented with dynamic stimuli capable of eliciting emotions.

Defining and quantifying mental flexibility might be one of the most challenging possibilities since it would enable affect dynamics to lend timing to affective states.

## Ethics statement

The studies involving humans were approved by the Ethic Committee of Istituto Auxologico Italiano IRCCS. The studies were conducted in accordance with the local legislation and institutional requirements. The participants provided their written informed consent to participate in this study.

## Author contributions

FB, AC, and PC elaborated the article rationale and revised deeply the related literature. FB and PC elaborated to formulas for the Markovian processes, created the delta indexes, computed the analyses, and the statistical model. FB wrote the first draft of the manuscript. All the authors revised the article and read the final version.
